# Demonstration of a Validated Direct Current Wearable Device for Monitoring Sweat Rate in Sports

**DOI:** 10.3390/s24227243

**Published:** 2024-11-13

**Authors:** Xing Xuan, Daniel Rojas, Isabel Maria Diaz Lozano, Maria Cuartero, Gastón A. Crespo

**Affiliations:** 1 UCAM-SENS, Universidad Católica San Antonio de Murcia, UCAM HiTech, Avda. Andres Hernandez Ros 1, 30107 Murcia, Spain; xxuan@ucam.edu (X.X.); jdrojas@ucam.edu (D.R.); imdiaz@ucam.edu (I.M.D.L.); mariacb@kth.se (M.C.); 2 Department of Chemistry, KTH Royal Institute of Technology, Teknikringen 30, SE-100 44 Stockholm, Sweden

**Keywords:** wearable sensor, sweat analysis, sweat rate, direct current method, microfluidic

## Abstract

Sweat rate magnitude is a desired outcome for any wearable sensing patch dedicated to sweat analysis. Indeed, sweat rate values can be used two-fold: self-diagnosis of dehydration and correction/normalization of other physiological metrics, such as Borg scale, VO2, and different chemical species concentrations. Herein, a reliable sweat rate belt device for sweat rate monitoring was developed. The device measures sweat rates in the range from 1.0 to 5.0 µL min^−1^ (2 to 10 µL min^−1^ cm^−2^), which covers typical values for humans. The working mechanism is based on a new direct current (DC) step protocol activating a series of differential resistance measurements (spatially separated by 800 µm) that is gradually initiated by the action of sweat, which flows along a customized microfluidic track (~600 µm in width, 10 mm in length, and 235 µm in thickness). The device has a volumetric capacity of ~16 µL and an acquisition frequency between 0.010 and 0.043 Hz within the measured sweat rate range. Importantly, instead of using a typical and rather complex AC signal interrogation and acquisition, we put forward the DC approach, offering several benefits, such as simplified circuit design for easier fabrication and lower costs, as well as reduced power consumption and suitability for wearable applications. For the validation, either the commercial sweat collector (colorimetric) or the developed device was performed. In five on-body tests, an acceptable variation of ca. 10% was obtained. Overall, this study demonstrates the potential of the DC-based device for the monitoring of sweat rate and also its potential for implementation in any wearable sweat platform.

## 1. Introduction

Sports science has experienced a revolution in recent years owing to the implementation of wearable technology that is capable of providing real-time monitoring for a variety of physiological indicators in sweat, such as ions, glucose, and lactate, among others [1,2,3,4,5]. Among those physiological indicators, sweat rate appears as a priority, given its uniqueness in tracing hydration/dehydration levels during sports practice and competition, but not restricted to these activities [6,7]. According to scientific reports, the effect of dehydration can occur with as little as a 1–2% loss of body weight and can become life-threatening when reaching 10% [8,9]. Therefore, early detection before reaching a dehydrated state is crucial. Notably, sweat rate values during physical activity have been reported to be in the range of 0.5–2.0 L/h (kg/h) [10], values obtained by means of the whole-body method. Also, it has been demonstrated that individualized hydration routines (linked to each person’s physiology) are more successful in preserving peak performance and averting consequences associated with dehydration [11,12].

Sweat rate can be estimated using a variety of methods classified as wearables and non-wearables. Regarding wearable devices, sweat patches are those that are fixed to the skin and gradually accumulate perspiration. The sweat rate is subsequently determined by analyzing the collected perspiration volume, therefore requiring external equipment for data processing [13,14]. There are wearable sensors that include built-in technology to enable a full on-body experience, with in situ monitoring and analysis of the sweat rate. To identify dynamic fluctuations in the sweat rate, a variety of sensing techniques based on colorimetry [15], capacitance [16], and resistance [17,18,19] have been employed. In contrast to the wearable approach, there are a bunch of non-wearable methods, such as urine analysis and subject weighing. In the case of urine analysis, the amount of sweat lost is estimated by measuring osmolality, specific gravity, protein, and potassium [20]. Then, weighing the person before and after the physical activity is a typical method conducted by physiologists to estimate the amount of perspiration lost. The sweat rate is then computed using the differential weight [20]. Overall, the precision of both non-wearable and patch-based methodologies is known to be not so high owing to their sensitivity to factors such as diuretic use, sample evaporation, and others. 

Wearable sweat rate sensors integrated with microfluidic devices have gained popularity in recent years because they prevent sample evaporation and enable real-time monitoring of sweat rate dynamics, even at small volumes of perspiration [13,21,22,23]. In essence, this technology allows for continuous sweat collection and the measurement of the corresponding rate [15,24,25,26,27]. Traditional manufacturing of microfluidic sweat rate sensors using soft lithography, as published by Hnin Yin Yin Nyein et al. [27], provides high precision but requires expensive instruments and involves complex fabrication processes. In contrast, the laser-cutting method used in this study offers simpler and more cost-effective fabrication, making it an attractive option for scalable production. 

The usage of sweat rate data in conjunction with other sweat-related parameters is very interesting. Some authors have made use of widely recognized sensor technologies, such as enzyme-based biosensors for metabolites and ion-selective electrodes for ions. Effectively, for these sensors to provide useful information on a person’s state of hydration and/or certain metabolic reactions during exercise, they frequently need to be integrated with sweat rate sensors for data correction and/or correlation [4,17,22,28].

Three readouts (resistance, capacitance, and colorimetry) have mainly been employed to monitor perspiration levels at the time of writing [15,16,17,18,19]. Additionally, Tomoaki et al. used traditional methods involving the measurement of the weight of collected sweat samples to calculate the sweat rate [29]. However, this approach is less promising because it requires external instruments for weight measurement. Among these, variations in resistance between two electrodes positioned within a microfluidic channel are well-liked since they are affordable and easy to fabricate, especially for developing on-body prototypes. Thus, it is possible to access quantitative measurements of sweat rate by measuring the electrical resistance across the channel and comparing the variations in resistance with the amount of sweat moving through it. Because of its high performance and ease of integration, this approach has found widespread usage in wearable devices, additionally providing real-time tracking capabilities. Moreover, it is appropriate for a variety of applications, including sports and healthcare [17,18,19,26]. Colorimetric-based sensors use chemicals (e.g., hydrogel-based ion-sensitive compounds or CoCl_2_) that react with certain substances (Cl^−^ or H_2_O) in the sweat to produce color changes. From the color change, the sweat rate can be estimated [15,30]. Also, sweat rate changes, inducing variations in the dielectric characteristics of certain systems, i.e., causing variations in the capacitance as the sweat moves through the connected channel, have been reported [16,31]. It is possible to quantify the volume of sweat and, consequently, the sweat rate by monitoring these variations.

Table S1 in the Supporting Information is provided to allow a clear view of the above-mentioned sweat rate sensors, which have been selected from the literature. The table includes critical parameters, such as analytical performance, concentration dependence, integration level, sampling, validation, and on-body assays. Only one colorimetry-based sensor underwent a full characterization, demonstrating its complete and reliable application for sports and even being commercially available [32]. Nevertheless, that device may be limited for users by the impracticality of taking pictures while training. Furthermore, some users, especially those with sensitive skin, may experience discomfort due to the orange dye remaining on the skin after utilization [33]. Then, the resistance-based sensors developed by Wei et al. and Yuan et al. provide beneficial features for the purpose of commercialization [17,19]. Wei and Yuan (also in this work) used a pulse-based sensing method, which allows the sensor to be independent of variations in ion concentration in sweat. In another work conducted by Lei Wei et al., their method involves copper electrodes in an admittance-based sweat rate sensor system that functions utilizing AC [34]. The main sensing mechanism includes detecting the rise in admittance of the electrodes as sweat travels through a microfluidic channel. One important feature of their technology is the ability to obtain high sensing precision while employing low-cost copper electrodes, making it a more inexpensive alternative to gold-based sensors. Notably, the main difference between our approach and the contributions by Wei, Yuan, and Wei relies on the direct current (DC) interrogation, which has never been reported for sweat rate measurements. Concretely, the DC step protocol is used to monitor voltage changes caused by sweat in microfluidic channels, while Wei, Yuan, and Wei employed alternating current (AC) techniques based on conductance. It is demonstrated herein that the DC method seems to lead in several aspects, as follows. The DC-based design simplifies the process of voltage conversion and phase synchronization. Also, the DC designs are naturally simple because current flows in only one direction, and this makes circuit design easier. Furthermore, the absence of conversion stages that contribute to inefficiencies in DC designs leads to reduced power consumption, which further increases its attractiveness [35]. The DC protocol results in the electrolysis of the sample and, nevertheless, does not affect the frequency of the voltage spikes in the device; hence, this does not affect the sweat rate calculation. This could lead to a change in the sample composition and, therefore, will enforce the use of different microfluidic channels for sweat rate and chemical sensing. In terms of microfluidic design (fabricated by laser cutting technology) and production, the developed device provides a reliable measurement method by catching sweat rates between 1.0 and 5.0 µL min^−1^. The device shows an acceptable acquisition frequency, ranging from 0.010 to 0.043 Hz, with a capacity of approximately 16 µL of sweat. By utilizing a simple DC method consisting of a single microcontroller unit (MCU), an operational amplifier (OP AMP) chip, a voltage regulator, and a minimal variety of resistors and capacitors, the sensor achieves acceptable results in terms of sweat rate values. Finally, the outcomes from the proposed validation method, accompanied by the negligible influence of the electrolyte concentration on the obtained sweat rate values, strongly indicate that the sweat rate belt device with the DC method is suitable for application in sports.

## 2. Experimental Section

**Reagents and instruments.** Analytical grade chloride salts of ammonium (CAS-12125-02-9), magnesium (CAS-7786-30-3), potassium (CAS-7447-40-7), and sodium (CAS-7647-14-5), as well as sodium carbonate (CAS-497-19-8), sodium bicarbonate (CAS-144-55-8), sodium hydroxide (CAS-215-185-5), ethanol (CAS-64-17-5), chlorophenol red (CAS-4430-20-0), and sodium phosphate (CAS-7558-79-4), were purchased from Sigma-Aldrich. The carbon ink was purchased from Henkel, Germany. All the solutions were prepared in 18.2 MΩ·cm doubly deionized water (Milli-Q water systems). Artificial sweat containing 60 mM NaCl, 6 mM KCl, 5 mM NH_4_Cl, 0.08 mM MgCl_2_, 2.6 mM NaHCO_3_, and 0.04 mM Na_2_HPO_4_ was used as the background in all the experiments for the characterization of the sweat rate belt device. The pH indicator (working from 4.0–8.0) is prepared with 0.1 wt% of chlorophenol red, 20 wt% ethanol, and 0.7 wt% of 1 M sodium hydroxide in Milli-Q water. 

The filaments for the 3D printing of the prototypes, TPU 95A and PLA transparent Ultimaker Material, were purchased from Ultimaker B.V., Netherlands. Electronics for signal (voltage) recording was designed through KiCAD and manufactured at PCBWay, China. Polyester sheets (thickness of 100 µm) to fabricate the screen-printed carbon electrode (for further modification to provide the sweat rate belt device) were acquired from RS online (Spain). Double adhesive tape (3M157559-ND) for integration of all the device parts and 3M 9473PC adhesive tape for microfluidics were purchased from Digikey, München, Germany. The SMD spring contact (ED9004-ND) for the connections between the electrodes and electronic board was purchased from Digikey, Spain. Macroduct device and iontophoresis kit for sweat collection were purchased from ELITechGroup (Spankeren, The Netherlands). The optimization of applied DC currents was performed with a potentiostat (Autolab, Metrohm, Switzerland). The CO_2_ laser system (Gweike Cloud Laser Cutter & Engraver with Rotary CO_2_ (50W) Pro was purchased from Gweike, Jinan, China. A syringe pump (TYD01-01, Baoding LFT Co., Baoding, China) and a peristaltic pump (ISM935C, ISMATEC, Tianjin, China) for calibration and off-body validation of the sweat rate belt device were purchased from VWR, Llinars del Vallés, Spain.

**Preparation of the electrodes for measuring resistance and integrating in the microfluidic cell.** The screen-printed carbon electrodes and the microfluidics were designed using Automatic Computer Aided Design 2024 (AutoCAD) software. The two electrodes for measuring the resistance were fabricated using a screen-printing method, while the electrodes’ substrate and mask were made from flexible mylar film. The mylar material was carefully cut to fit into the intended electrode design using a Silhouette cutter. To ensure conductivity and electrode adhesion, carbon paste was screen-printed onto the mask and then cured in the oven for an hour at 100 °C (Figure S1). Simultaneously, double adhesive tape was used to create the microfluidic channel, resulting in a leak-proof platform. The dimensions of the channel in the CAD microfluidic design are 400 µm for width, 10 mm for length, and 235 µm for thickness of double adhesive tape. The CO_2_ laser instrument was utilized to cut (laser precision of 25 µm) the double adhesive tape in order to form microfluidic channels with the following optimized parameters: power of 20 W (40%), speed of 40 mm/s, and Z-axis of −10 mm, respectively (Figure S2). The actual width of the channel was measured using an optical microscope (Nikon Eclipse Ti2, Nikon, Tokyo, Japan), shown in Figure S3 with the 630 µm, which is caused by the burning during the cutting process. Additionally, a 0.5 cm^2^ area of the skin (4 mm radius circle) is chosen as the sweat-gathering area for the sweat rate belt device. To prevent any leaks of liquids inside the microfluidic channel. The two electrodes for measuring resistance and microfluidic channels were carefully integrated. The capacity of the device (15.7 ± 0.8 µL of sweat sample) was estimated using a syringe pump. 

**Design of the hardware and software.** The electrical circuits were designed with KiCAD 7.0, introducing several key components such as signal processing, amplification, and analog-to-digital conversion. Afterwards, a printed circuit board (PCB) layout was made, and the final PCBs were produced by a manufacturing company (PCBWAY, Shenzhen, China). Segger Embedded Studio 7.30, a software platform for embedded systems especially used for the Nrf52832 chip, was used to write the firmware for the microcontroller unit (MCU) integrated within the PCB. This firmware, which forms the core of the sweat rate belt device, was able to collect the voltage outputs between the two electrodes and perform the signal transmission. Bluetooth technology was used to connect the PCB and external receiver wirelessly. The software mobile application was built with Android Studio. The program displayed real-time output voltage signals obtained from the sweat rate belt device. Additionally, the program permitted the storage of the date and time of the experiment, which makes it possible to properly analyze sweat rates from different tests and subjects. 

**Validation protocol using a commercial iontophoresis device.** The validation methodology consists of two subsequent steps. The first step was indeed the stimulation of sweat by a commercial iontophoresis device, following the guidelines of the product (applied current of iontophoresis: 1.5 mA with current density of density of 240 μA cm^−2^; pilocarpine concentration of gel: 0.5%) [36], whereas the second one related to the determination of the sweat rate by either the sweat rate belt device or the Macroduct (ELITechGroup, Spankeren, The Netherlands) sweat collector (via length-based analysis). 

Briefly, in the step of stimulating sweat, commercial hydrogels containing pilocarpine were positioned onto the cathodic and anodic electrodes of the Macroduct system, ensuring a close distance between them. To prevent any injury, a drop of water to the skin was applied before positioning the electrodes. Subsequently, a 1 mA current was applied through the cathodic and anodic electrodes for a duration of 10 min. Once completed, all electrodes were removed, and the skin was cleaned using either cotton or paper. The skin region where the cathodic electrode was placed was ready for the placement of either the sweat rate belt device or the Macroduct sweat collector for sweat rate analysis.

In the first case, the sweat rate belt device was positioned on such an area, and once the device was activated, it began sending data via Bluetooth connection to the custom-built smartphone application. The signal output was expressed in voltage and converted to sweat rate units according to the methodology herein developed (see below). For the Macroduct sweat collector, markings were made on it to identify the sweat positioning coinciding in time with each peak received from the sweat rate belt device. The sweat rates from the Macroduct sweat collector were calculated using the length measurements of the sweat advancement within the tubing inside the collector. The entire procedure is illustrated in Figure S4 for clarity. The validation of the measurements provided by the sweat rate belt device was carried out by investigating the matching with the Macroduct sweat collector via uncertainty estimations. The sweat rate belt device’s capabilities were thoroughly evaluated owing to this validation process, which laid out the basis for its use in future practical situations.

## 3. Results and Discussion

**Figure 1:**
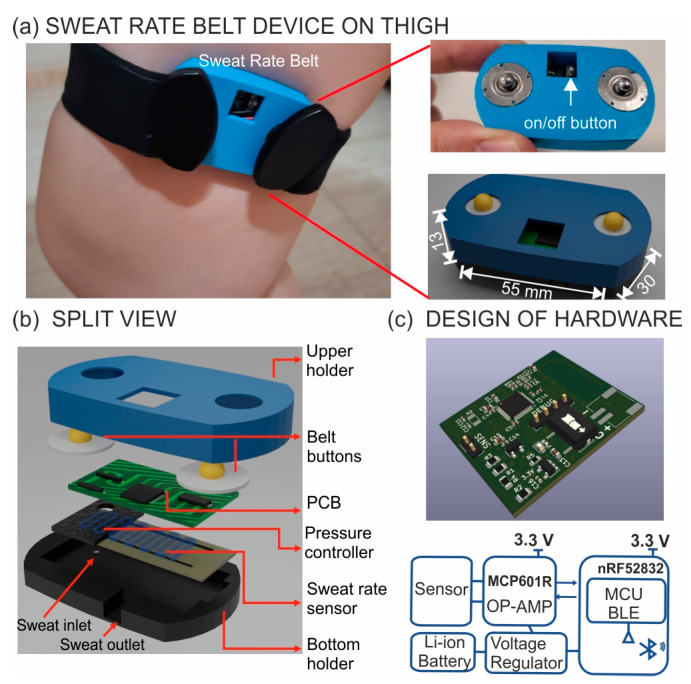
(**a**) Image of sweat analysis with the developed sweat rate belt device fastened to the thigh. Magnifications of the upper and bottom parts of the device are included. (**b**) Details of all the layers included in the device. (**c**) 3D view of the printed circuit board (PCB) together with the circuit block diagram.

**The sweat rate belt device.** Figure 1a provides real images of the developed sweat rate belt device functioning on a thigh. The complete device was designed with a size of 13 × 55 × 30 mm and a weight of only 7.0 g, including the battery. This small configuration was essential for wearability and user convenience. The schematic of the layering of the sweat rate belt device is shown in Figure 1b. Then, the printed circuit boards are depicted in Figure 1c, with a size of 27 × 33 × 1 mm. The system block diagram (Figure 1c) illustrates each component while furnishing a perspective of the signal flow and interactions between the various components.

**Figure 2:**
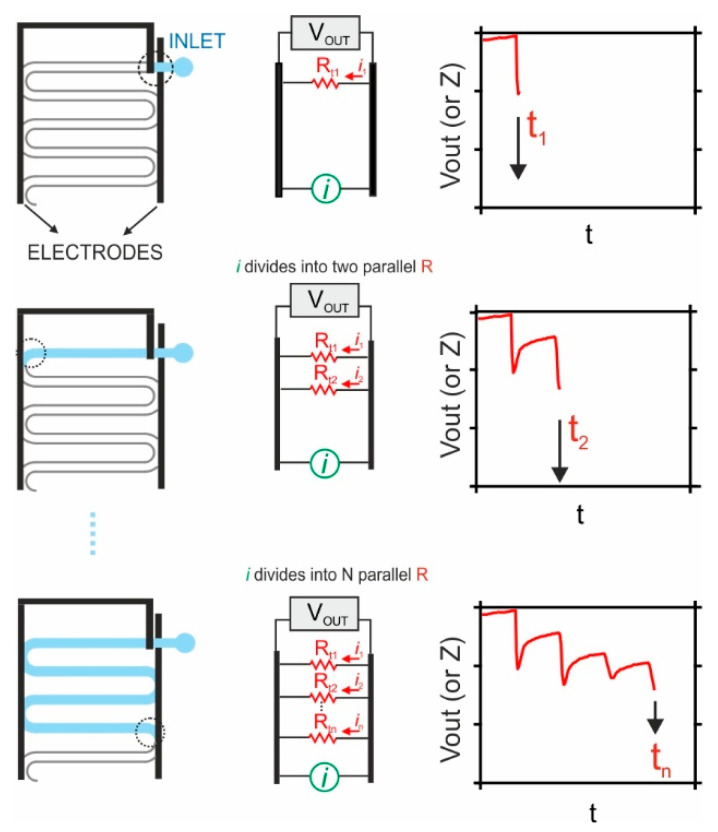
Diagram illustrating the sensing mechanism in the sweat rate belt device. It is indicated how perspiration rate is detected and the obtention of the pertinent information. The *i* and V_OUT_ (or Z) represent the applied current and voltage output, respectively. The different R_tn_ indicate the parallel resistances between two electrodes at t_n_.

**The sensing mechanism of the sweat rate belt device.** The working mechanism of the sweat rate belt device is depicted in Figure 2. The sweat enters the fluidic cell across the inlet and flows continuously and gradually through the fluidic channel, which has a capacity of approximately 16 µL. While the sweat flows across the channel, electrical connections between two conductive electrodes (black) are established because the sweat acts as a “short circuit” media. The resistance of these electrical connections can be measured either using an AC method, as reported previously in the literature, or using a DC method, as we propose in the present manuscript. Each time a new electrical connection is created, a new resistor connected in parallel is added to the total resistance of the system. Therefore, the main analytical signal providing information related to the sweat rate is the frequency at which each of the electrodes is reached by the liquid (sweat), regardless of whether the change is monitored with a DC or AC method. 

As follows, we expand the working mechanism considering the proposed DC method. Essentially, a fixed DC current is applied to the electrodes, and the voltage between these is monitored. When sweat advances through the serpentine channel, it successively creates connections between the two electrodes, forming parallel resistances (from 2 to N) in the system. Thus, each time of connection reduces the overall resistance of the entire system. According to Ohm’s Law, this resistance is directly proportional to the output voltage. Then, as the sweat bridges the gap between the electrodes, the current is distributed across the newly formed parallel resistances, resulting in a decrease in the voltage output. As the number of parallel resistance connections (nodes) increases, the change in total system resistance becomes smaller with each additional node, resulting in reduced voltage changes. This voltage degradation may limit the sensitivity of the device while more nodes are added.

The initial spike (*t*_1_) denotes the exact instance at which sweat begins to flow through the microfluidic channel. Subsequently, a stable background voltage occurs until a new (sweat-based) connection appears in the microfluidic channel, leading to an immediate decrease in the voltage signal at a certain time (*t*_2_) due to the sudden changing in resistance between the two electrodes. This process allows for the calculation of the differential time (Δ*t*) between voltage signal spikes ($t_{n}-t_{n-1}$), which is the base for determining the sweat rate. For such a purpose, we proposed the use of Equation (1):

(1) $$SR=\frac{S}{\left(\Delta t-b_{0}\right)*A}$$

where *SR* represents the sweat rate (µL min^−1^), while S (s µL min^−1^) and *b*_0_ (s) are the slope and intercept of the pre-calibration parameters, respectively (as explained below and illustrated in Characterization section). Δ*t* (s) is the time difference between two voltage signal spikes, and *A* represents the area of sweat gathering (0.5 cm^2^).

**Figure 3:**
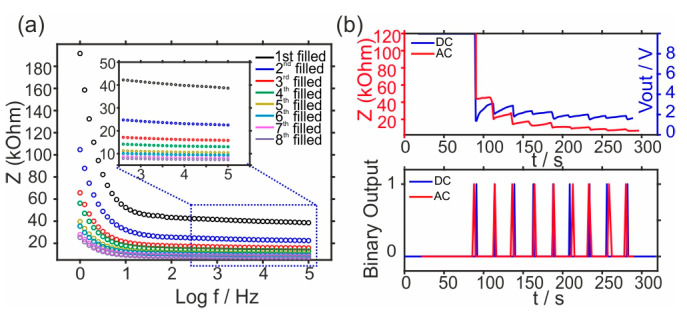
(**a**) EIS measurements performed across a frequency range from 100 kHz to 1 Hz at the different stages of the device filling with artificial sweat. (**b**) The outcomes of the two techniques: the AC approach (depicted in red, indicating impedance output) and the DC method (depicted in blue, indicating voltage output). In the AC method: DC base–voltage of 0 V and AC voltage–amplitude of 10 mV in the form of a sine wave were applied.

**Comparison of DC and AC methods.** Figure 3a shows the signals obtained from electrochemical impedance spectroscopy (EIS) at the different stages of the device filling and, hence, increasing the number of resistors’ connections (See Figure 2). The purpose of this experiment was to assess the impedance output signal when artificial sweat fills several channels. In theory, the impedance is expected to decrease as the number of occupied channels grows, owing to the parallel resistor arrangement of the channels. It was observed that the impedance values between 1 kHz and 100 kHz mainly exhibit a close-like resistive behavior, with a phase angle near 0 (see Figure S5), and this confirms that the capacitive processes (e.g., capacitive effects, including double-layer capacitance at the electrode–sweat interface) are negligible, resulting in a stable resistive response within this frequency range. This results in a predominantly resistive reaction, as evidenced by the almost 0 phase angle. 

To compare both AC and DC methods, both the *Z* vs. *t* and *V* vs. *t* curves were plotted together in Figure 3b. The measurements were conducted by applying a fixed DC current of 1.6 µA while recording the V output (DC method) or an AC voltage of 10 mV at a frequency of 10 kHz (AC method), both while the device was being filled at 5 µL min^−1^. Importantly, the applied current (1.6 µA) was well below the discomfort level (the discomfort level is considered as under 1 mA), with no reported sensations from participants during the test. The application of the step-current protocol may result in some disadvantages, such as side electrochemical reactions and electrode polarization. This can indeed be the reason why the DC method time transient signal was slightly less stable compared to the AC method. Nevertheless, when the binary output of the signal was compared, no significant differences were found. Moreover, the results demonstrated that the AC approach had an average time of 23.0 ± 2.5 s, while the DC method presented an average time of 23.0 ± 1.6 s between spikes, being both perfectly comparable.

Notably, the voltage generated between the electrodes using the DC method may generate side electrochemical reactions. We performed some experiments to assess whether this affects the sweat rate measurements. Thus, the sweat rate belt device was loaded with a NaCl solution containing a pH indicator. After applying the DC current continuously for 30 min, photos were taken at 0, 1, 5, and 30 min. As shown in Figure S7, a color change was produced in both the anode and cathode over a long time. This confirmed that redox reactions are indeed taking place on the electrodes’ surfaces. Presumably, H_2_ generation occurs in the cathode, increasing the pH due to the H^+^ consumption, whereas O_2_ evolution happens in the anode, with the respective pH decrease due to H^+^ generation. Nevertheless, the rates at which the reactions occurred were relatively low due to the long time required to observe a change in the pH. In addition, no bubble generation was observed, which may produce microchannel clogging. We repeated the experiment with artificial sweat (Figure S7), and pH changes were not observed over time. This ensured that, when using artificial sweat (or human sweat), the pH of the sample remains constant and unaffected by the electrolysis owing to its natural buffer capacity. 

**Figure 4:**
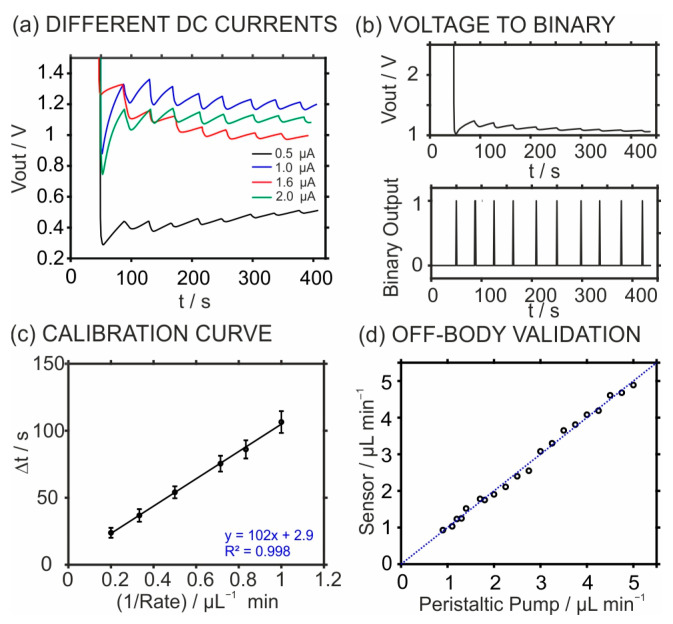
(**a**) Variation of the voltage output at different DC currents and the (**b**) signal processing method used for data conversion by transforming a voltage signal into a binary output with the injection rate of 2.5 µL/min. (**c**) Calibration curve for the sweat rate belt device. (**d**) Off-body validation of the device by measuring the sweat rate imposed by a peristaltic pump.

**Characterization of the sweat rate belt device.** We optimized the applied DC by selecting four distinct current levels, spanning from 0.5 to 2.0 μA. The minimal differences in voltage (ΔV) between the two peaks, as depicted in Figure 4a, were measured to be 11.3 mV ± 2.1, 32 mV ± 14.9, 31 mV ± 8.5, and 24 mV ± 8.2 for the currents of 0.5, 1.0, 1.6, and 2.0 μA, respectively. Importantly, a larger minimum ΔV value enhances data processing by yielding a higher signal-to-noise ratio. Both 1.0 μA and 1.6 μA currents resulted in high ΔV values of around 32 mV. Applied currents in the range from 1 μA to 2 μA did not represent any substantial change. While according to Ohm’s Law, increasing the DC current should result in a proportionate increase in the minimum ΔV, practical results were found not to follow this trend. This may be attributable to voltage output changes in each measurement within the small applied current range. Applying a higher current could indeed result in a larger ΔV, but it may also introduce the risk of significant electrolysis during the measurement. To balance the signal intensity and stability ΔV, a current of 1.6 µA was selected for further experiments, as this level of ΔV meets the requirements of the on-body application. 

Figure 4b depicts the signal processing herein used to transform the raw voltage signal by flowing artificial sweat into a binary output, similar to a methodology reported elsewhere [17]. Briefly, the algorithm (Script S1) detects potential spikes by applying two key parameters: the first voltage drop (with changes less than 10% of the minimum delta V considered as noise) and the interval time (calibrated from the sensor). Within each time interval, the algorithm identifies the first voltage drop, assigns it a binary output of 1, and sets all other points to 0 (one example is shown in Figure S8). This process is repeated across intervals, and the time values corresponding to the binary 1 outputs are used to calculate the time difference (Δt), which is then substituted into equation 1 to obtain the SR value. Notably, the collection of distinct time periods between spikes, a critical parameter needed for the sweat rate calculation using the developed device, is easily achieved by this conversion. Figure S9 shows the system response to flow rates (1, 1.2, 1.4, 2, 3, and 5 µL/min) of artificial sweat generated by the syringe pump. The binary output shows distinct times between successive peaks, which were directly related to the flow rates. Notably, because of the faster channel filling speed at the higher flow rates, time intervals were shortened. For these flow rates, the average times needed were 104.8 ± 5.5, 87.3 ± 6.4, 75.7 ± 5.0, 51.3 ± 2.5, 34.6 ± 2.3, and 21.3 ± 2.4 s for 1, 1.2, 1.4, 2, 3, and 5 µL/min, respectively. 

From the data obtained at the different rates and considering Equation (1), the inverse of the rate versus Δt was plotted to generate the calibration graph. Figure 4c shows the average linear fitting observed for three similar sweat rate belt devices in the range from 1 to 5 µL/min. Considering all the points from the triplicate experiment (N = 3), we obtain an average slope of 102 ± 9 s µL^−1^ min and an intercept of 3 ± 5. Importantly, the sweat rate belt device covers the normal perspiration rate that is expected during exercise [18,37]. Thus, this calibration provides an appropriate basis for sweat rate measurements in later on-body tests. 

Next, a peristaltic pump was employed as an alternative injection method of the artificial sweat to perform a preliminary off-body validation of the sweat rate belt device. The correlation between the rates imposed by the peristaltic pump and those measured by the device (calculated by means of a previous calibration graph using the syringe pump) is shown in Figure 4d. The correlation included twenty distinct flow rates and displayed a slope of 0.99 with uncertainty of 0.02, an intercept close to 0.03 with uncertainty of 0.05, and a Pearson coefficient of 0.996, therefore demonstrating the device’s excellent reliability. Moreover, an average difference of 0.10 ± 0.04 µL/min was found between the rate values of both techniques. Once more, the outcomes indicate that the sweat rate belt device is, in principle, suitable for sweat rate monitoring in future on-body tests.

**Figure 5:**
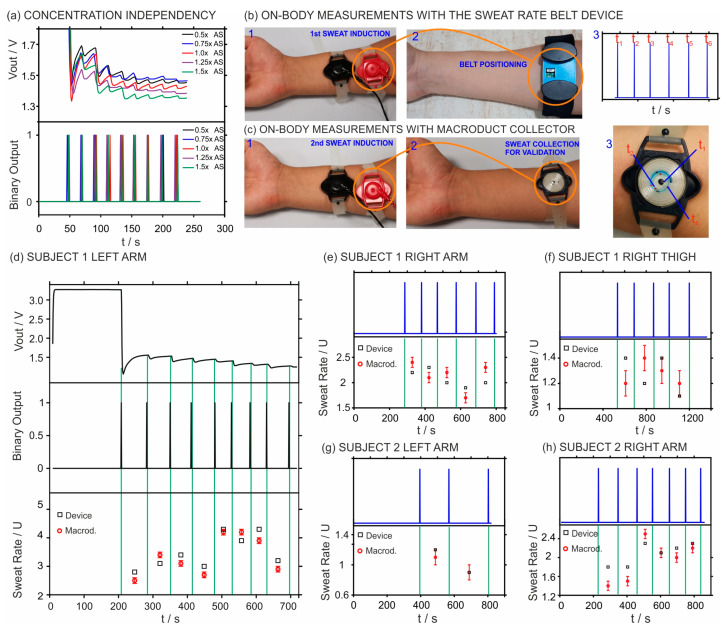
(**a**) Response of the device to five samples containing different ionic strengths of the artificial sweat: 0.5X, 0.75X, 1.0X, 1.25X, and 1.5X at an injection rate of 5 µL min^−1^. (**b**) Images corresponding to sweat induction with the Madroduct iontophoresis system (step 1), measurements with the sweat rate belt device (step 2), and the observed binary output (step 3). (**c**) Images corresponding to sweat induction with the Madroduct iontophoresis system (step 1), measurements with the Macroduct sweat collector (step 2), and the sweat labeling performed to obtain the sweat rate at specific times (step 3). (**d**) On-body results from the left arm of Subject 1: voltage and the corresponding binary output, as well as the calculated sweat rates with both the sweat rate belt device and the Macroduct collector. In this latter plot, the *U* in the *y*-axis represents the unit of µL min^−1^ cm^−2^, and the red circles and black rectangles represent the average perspiration rate provided by the Macroduct collector and the sweat rate belt device, respectively. (**e**,**f**) On-body results (binary output and sweat rates) from the right arm and right thigh of Subject 1. (**g**,**h**) On-body results (binary output and sweat rates) from the right and left arms of Subject 2.

**Investigation of concentration independence on the sweat rate output.** Investigation of concentration independence on the output was then performed to evaluate the concentration influence with five different concentrations. The concentration of each chemical in the prepared artificial sweat was set as X, and the five test solutions were set to be 0.5X, 0.75X, 1.0X, 1.25X, and 1.5X, which cover the range in human sweat [38]. The sweat rate was fixed at 5.0 µL/min for both samples, and each one was measured three times using the same sweat rate belt device. The voltage output signals obtained from these five samples were converted into binary outputs, and the results are presented in Figure 5a. We observed an average of 22.0 ± 1.0 s between the spikes provided by each concentration, which corresponds to a fluctuation of ca. 0.23 µL/min at an injection rate of 5.0 µL/min, i.e., a variation of 5%. To understand whether such a variation is a consequence of the electrolyte concentration change or just an inherited characteristic of the device, we evaluated the triplicate measurements for both concentration levels. Average responses of 21.9 ± 0.8 s (corresponding to 0.18 µL/min at the injection rate of 5.0 µL/min), 22.0 ± 0.9 s (corresponding to 0.20 µL/min at the injection rate of 5.0 µL/min), 20.0 ± 0.8 s (corresponding to 0.18 µL/min at the injection rate of 5.0 µL/min), 22.3 ± 1.4 s (corresponding to 0.32 µL/min at the injection rate of 5.0 µL/min), and 21.8 ± 1.0 s (corresponding to 0.23 µL/min at the injection rate of 5.0 µL/min) were observed for artificial sweat of 0.5X, 0.75X, 1.0X, 1.25X, and 1.5X, respectively. Notably, the variations (between 2% and 6%) found suggest that changes in electrolyte concentration have a negligible impact on the performance of the sweat rate belt device. Moreover, in our experiments, we have used a very wide range of electrolyte concentrations, and such a drastic difference is not expected to occur in a subject when monitored with the developed sweat rate belt device.

**On-body tests using the iontophoresis system: investigation of the validation strategy of the sweat rate belt device.** Subsequently, we run a series of on-body tests based on the use of both the developed sweat rate belt device and the Macroduct sweat collect after applying iontophoresis for sweat stimulation in the subject. The protocol is detailed in the Experimental Section and was considered in the ethical permit CE062308 dated 30 June 2023 (UCAM Ethics Committee).

To have a clean surface, the participant’s skin was first cleaned with ethanol and water before applying iontophoresis. The iontophoresis was then applied for 10 min [36]. Then, the sweat rate belt device was positioned at the cathodic electrode site, as shown in Figure 5b. The voltage signal was recorded by the device, showing clear spikes that were transformed first into the binary output and later into perspiration rates by means of a previous calibration graph. 

A similar procedure was followed to obtain the sweat rate based on the Macroduct collector, i.e., the same experimental conditions were replicated before positioning the Macroduct sweat collector in the area coinciding with the cathodic electrode (Figure 5c). Then, the sweat flowing inside the collector was labeled at the same times as the spikes that were acquired from the sweat rate belt device (Figure 5c). The length for each acquired point and corresponding times were used to calculate the sweat rates inside the sweat collector for further comparison with the data provided by the sweat rate belt device.

The left arm of *Subject #1* was selected to perform the on-body test following the above-mentioned procedure, resulting in the first point suitable for sweat rate acquisition occurring after 200 s from finishing the iontophoresis. This is mainly linked to the time that the skin needs to start sweating after the delivery of pilocarpine in the iontophoresis process. This time may range from 1 to 5 min or even longer, being influenced by individual variances in the sweat collection technique and skin physiology [39]. As shown in Figure 5d, the raw voltage output signal was converted to a binary output signal. Then, by measuring the time between each spike, eight intervals of 74, 68, 61, 69, 48, 54, 48, and 65 s were obtained. The calibration curve obtained for the sweat rate belt device was used to determine the sweat rates for each of the intervals, resulting in rates of 1.4, 1.5, 1.7, 1.5, 2.2, 1.9, 2.2, and 1.6 µL/min. Counting on the sweat collection area (0.5 cm^2^), the rates were measured to be 2.8, 3.1, 3.4, 3.0, 4.3, 3.9, 4.3, and 3.2 µL min^−1^ cm^−2^, as shown by the black circles in Figure 5d. 

Before performing the on-body test in the left arm of *Subject #1* with the Macroduct collector, a syringe pump with fixed rates (1–5 µL min^−1^) was used to evaluate the inaccuracy of the system. The test was accomplished on three different Macroduct collectors going through six similar trials. The results are shown in Table S2 in the Supporting Information for samples collected at one-minute intervals for each injection rate. In essence, the rate imposed by the pump was measured in the Macroaduct collector, and the results were compared. Within the range from 1 to 5 µL min^−1^, the average difference between the rate provided by the syringe pump and that calculated in the collector was 0.1 ± 0.06 µL min^−1^ (absolute value), which is indeed an acceptable result aligned to that observed for the sweat rate belt device. In the on-body tests using the Macroduct collector, the sweat rate within each interval was measured using Equation (2).

(2) $$v=\frac{L\;A_{1}}{t\;A_{2}}$$

where *t* (min) is the time interval between two peaks obtained from the sweat rate belt device, *A*_1_ (cm^2^) is the inner area of the tubing inside the collector, *v* (µL min^−1^ cm^−2^) is the perspiration rate, and A_2_ (cm^2^) is the sweat collection area. Thus, the calculations made use of the constant parameters *A*_1_ = 0.32 mm^2^ and *A*_2_ = 71.5 mm^2^. The red circles in Figure 4d represent the resulting perspiration rates, which averaged to be 2.5, 3.4, 3.1, 2.7, 4.2, 4.2, 3.9, and 2.9 µL min^−1^ cm^−2^. Then, the average discrepancy between the sweat rate belt device and the Macroduct collector-based results was found to be 0.3 ± 0.1 µL min^−1^ cm^−2^. 

The positioning of the sweat rate belt device in the right arm of *Subject #1* was also investigated in the on-body tests. In this case, the first binary spike was obtained at ca. 286 s from finishing the iontophoresis. Figure 5e presents the binary output and the sweat rates measured with the device and the collector.

A total of five intervals were recorded, with durations of 94, 89, 106, 111, and 104 s, respectively. The sweat rates obtained from the sweat rate belt device were determined to be 2.2, 2.3, 2.0, 1.9, and 2.0 µL min^−1^ cm^−2^, as shown by the black squares. The rates that were observed from the Macroduct were 2.4, 2.1, 2.2, 1.7, and 2.3 µL min^−1^ cm^−2^, as shown by the red circles. The average difference between the results provided by both techniques was found to be 0.2 ± 0.1 µL min^−1^ cm^−2^, in the same order as in the left arm. Nonetheless, the right arm’s sweat rates were found to be lower than those of the left arm. Such a difference could be caused by the fact that the measurements were not accomplished on the same day or due to typical body-related variations already mentioned in the literature [39].

Perspiration rate monitorization in the right thigh of *Subject #1* was additionally accomplished, revealing a longer time (ca. 554 s after iontophoresis) to obtain the first binary spike suitable for the sweat rate calculation value than in the arms. Notably, while applicable in any part of the body, the Macroduct guidelines recommend its use in the arms to maximize the effectiveness of sweat generation. Accordingly, a longer time for sweat generation in the thigh is expected. As shown in Figure 5f, four intervals of 154, 180, 145, and 186 s were recorded. The sweat rate belt device provided the following results: 1.4, 1.2, 1.4, and 1.1 µL min^−1^ cm^−2^. The corresponding sweat rate readings from the Macroduct collector were 1.2, 1.4, 1.3, and 1.2 µL min^−1^ cm^−2^. The average differences between the sweat rate belt device and the Macroduct values were found to be 0.2 ± 0.1 µL min^−1^ cm^−2^ once more. The rates of sweating on the thigh were confirmed to be lower than those on the arms.

Next, perspiration rates in the left and right arms of a second subject (*Subject #2*) were studied. In the left arm (Figure 5g), the first binary spike was obtained at 395 s, while in the right arm (Figure 5h), this was obtained at 230 s after applying iontophoresis. In the left arm, only two possible data points were obtained (at 173 and 235 s). The very low level of sweating noted in this particular case led to the limitation of the dataset. These two points were associated with sweat rate values of 1.2 and 0.9 µL min^−1^ cm^−2^, as determined by the sweat rate belt device, and 1.1 and 0.9 µL min^−1^ cm^−2^, as observed in the Macroduct collector. Once more, the differences found between the two approaches were not noticeable (0.07 ± 0.06 µL min^−1^ cm^−2^). In the right arm, a larger dataset was obtained, consisting of six recorded intervals at 117, 113, 92, 101, 96, and 90 s. The sweat rate belt device provided perspiration rates of 1.8, 1.8, 2.3, 2.1, 2.2, and 2.3 µL min^−1^ cm^−2^, whereas the collector presented rates of 1.4, 1.5, 2.5, 2.1, 2.0, and 2.2 µL min^−1^ cm^−2^. The average difference between the results revealed by the sweat rate belt device and collector was 0.2 ± 0.1 µL min^−1^ cm^−2^, i.e., coinciding with the observations in other body parts and subjects.

Notably, the comparison of the results from the left and right arms in *Subject #2* pointed out strong regional differences in perspiration rates, as already mentioned for *Subject #1*. Therefore, it is important to account for not only individual variances but also regional ones when physiological meanings are pursued. Regarding the use of iontophoresis to induce skin sweating, the method has different impacts on different body areas, depending on things like skin physiology and thickness. After iontophoresis, the skin usually starts to sweat after 1 to 5 min (even longer), though this onset time varies depending on the agent concentration and the hydration level of each subject [39].

**Figure 6:**
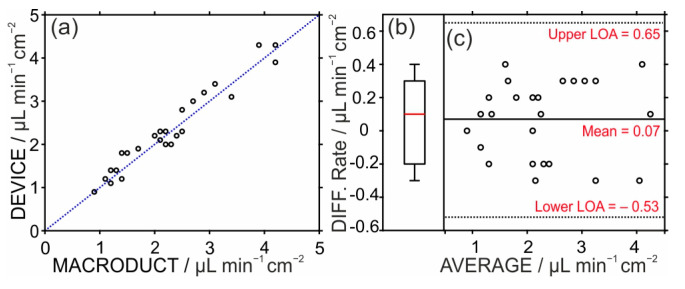
(**a**) Correlation plot between the on-body data provided by the sweat rate belt device and Macroduct sweat collector. Blue dashed line represents the ideal correlation case. (**b**) Box plot the on-body data provided by the sweat rate belt device and Macroduct sweat collector. (**c**) Bland–Altman plot depicting the agreement between the two methods.

**Statistical analysis of the outcomes obtained in the on-body tests.** Figure 6a depicts the plot of the correlation between the results obtained with the sweat rate belt device and those observed with the Macroduct sweat collector, utilizing a total of 25 data points (collected in Table S3 in the Supporting Information). In the figure, the blue dashed line represents the ideal correlation case, i.e., linear relationship with slope = 1 and an intercept = 0. The linear fitting of the data revealed a slope of 0.981 ± 0.01 and intercept of 0.11 ± 0.06, which are indeed close to the ideal behavior. Moreover, the Pearson coefficient (0.97) confirmed the positive correlation between both methods.

To determine and interpret the average difference between the results shown by the sweat rate belt device and the Macroduct collector at a 99% confidence interval, a dependent sample *t*-test was used. The *t*-score (N = 25) was 1.12, which was smaller than the critical theoretical value of 2.79, indicating no significant differences between both methods. The different values were observed to be around zero in the boxplot (Figure 6b). Moreover, the data showed results with a median of 0.1 µL min^−1^ cm^−2^, first quartile of −0.2 µL min^−1^ cm^−2^, and third quartile of 0.3 µL min^−1^ cm^−2^. The Bland–Altman plot, which shows the variance between two devices against their average values (Figure 6c), provided a more thorough analysis of the agreement between individual data, additionally detecting possible trends and inconsistencies. The error distribution in the results did not vary over the range from 0.9 to 4.3 µL min^−1^ cm^−2^, suggesting that variations were unrelated to the recorded sweat rates. However, the determined lower and upper limits of agreement (LoA), which are set at −0.53 and 0.65, were found to be slightly wide. This may be caused by the variations presented in distinct body parts and different subjects. Overall, all the statistical results highlighted the adequate accuracy of the developed sweat rate belt device when compared with the commercially available Macroduct collector. Notably, this is later found in the literature to obtain the sweat rate and also to validate new techniques [16,40].

## 4. Conclusions

This investigation demonstrates a new sweat rate belt device to successfully measure perspiration rates in the range from 1.0 to 5.0 µL min^−1^, which is typical during the practice of physical activity. The established design, fabrication approach, and utilization of a much simpler DC method instead of traditional AC methods resulted in the device achieving acceptable results in sweat rate measurements, displaying a noticeable between-device reproducibility. Regarding the readout utilized for the sweat rate observations, this is based on voltage variations between two electrodes when sweat is flowing along them. Then, such differences are converted into a binary language that allows for the calculation of the sweat rate using an established algorithm mainly based on the times at which binary spikes appear while naturally accompanying the subject perspiration. The device was rather accurate in on-body tests, considering 25 data points observed with the developed sweat rate belt device and the commercially available Macroduct collector after applying iontophoresis to different subjects and different body regions. The independence of the sweat rate measurements from electrolyte concentration variations, even in dynamic conditions, is an advantage of the sweat rate belt device compared with other devices previously published in the literature. With a capacity of approximately 16 µL and an acquisition frequency ranging from 100 s to 23 s, the device enables capturing of perspiration rates in very low volumes of sweat. While the sweat rate belt device displays reliable performance in sweat rate monitoring, the current system confronts constraints in terms of measurement intervals and node count. Future advancements will focus on refining the microfluidic channel architecture and electrode shape to maximize measurement frequency and decrease these restrictions.

## Data Availability

Data are contained within the article.

## Supplementary Materials

The following supporting information can be downloaded at: https://www.mdpi.com/article/10.3390/s24227243/s1, Figure S1. Layering of the screen-printed electrodes included in the sweat-rate-belt-device. Figure S2. Schematics of the cutting process used to develop the microfluidic channel using the CO_2_ laser system. Figure S3. The photo shows the width of microfluidic channel (scale bar is 250 μm). Figure S4. Illustration of the steps included in the method for sweat rate measurement using the Macroduct collector. Figure S5. The Electrochemical Impedance Spectroscopy (EIS) performed across a frequency range of 100 kHz to 1 Hz at the different stages of the device filling with artificial sweat. Figure S6. The Impedance value with number of channels filled at 10 kHz. Figure S7. Photographs at 0, 1, 5, and 30 minutes in artificial sweat (left) and 0.1 M NaCl (right) after continuously applying the DC current in the system. Figure S8. Example of data conversion by transforming a voltage signal into a binary output. Figure S9. Variation of the binary output at different sample rates. Δ*t* represents the average time difference between two signal spikes. Table S1. Comparison of the sweat rate sensors already reported in the literature and that herein developed. Table S2. The error estimation of the Macroduct device using syringe pump. Samples were collected at one-minute intervals during each injection. Table S3. Validation of on-body sweat rate measurements observed with the sweat-rate-beltdevice. The results are compared with the Macroduct sweat collector. Script S1. MATLAB script of the algorithm for convert time vs. voltage to time vs. binary.
