# Supplementary material for: Demonstration of a Validated Direct Current Wearable Device for Monitoring Sweat Rate in Sports
Source: Sensors (Basel). 2024 Nov 13;24(22):7243. doi: 10.3390/s24227243 (PMC11598451; doi:10.3390/s24227243)
Supplement: Supplementary file 1 [file sensors-24-07243-s001.zip › sensors-3254029-supplementary.pdf]

Supporting Information for:

Wearable Device for Continuous Sweat Rate Monitoring in Sports

Xing Xuan[1], Daniel Rojas[1], Isabel Maria Diaz Lozano[1], Maria Cuartero[1,2], and Gastón A. Crespo[1,2,*]

[1]*UCAM-SENS, Universidad Católica San Antonio de Murcia, UCAM HiTech, Avda. Andres Hernandez Ros 1, 30107, Murcia, Spain.*
[2]*Department of Chemistry, KTH Royal Institute of Technology, Teknikringen 30, SE-100 44, Stockholm, Sweden.*
*Corresponding authors: gacp@kth.se

Table of Contents

1. Figures 2

Figure S1. Layering of the screen-printed electrodes included in the sweat-rate-belt-device 3

Figure S2. Schematics of the cutting process used to develop the microfluidic channel using the CO2 laser system 3

Figure S3. The photo from microscope showing the width of microfluidic channel 4

Figure S4. Illustration of the steps included in the method for sweat rate measurement using the Macroduct collector 4

Figure S5. The Electrochemical Impedance Spectroscopy (EIS) performed across a frequency range of 100 kHz to 1 Hz at the different stages of the device filling with artificial sweat 5

Figure S6. The Impedance value with number of channels filled at 10 kHz. 5

Figure S7. Photographs at 0, 1, 5, and 30 minutes in 0.1M NaCl after continuously applying the DC current in the system 6

Figure S8. Example of data conversion by transforming a voltage signal into a binary output. 6

Figure S9. Variation of the binary output at different sample rates. Δt represents the average time difference between two signal spikes 7

2. Tables 8

Table S1. Comparison of the sweat rate sensors already reported in the literature and that herein developed 8

Table S2. The error estimation of the Macroduct device using syringe pump. Samples were collected at one-minute intervals during each injection 8

Table S3. Validation of on-body sweat rate measurements observed with the sweat-rate-belt-device. The results are compared with the Macroduct sweat collector 9

3. MATLAB script 10

Table S1. MATLAB script of the algorithm for convert time vs. voltage to time vs. binary. 10

3. References 11

1. Figures

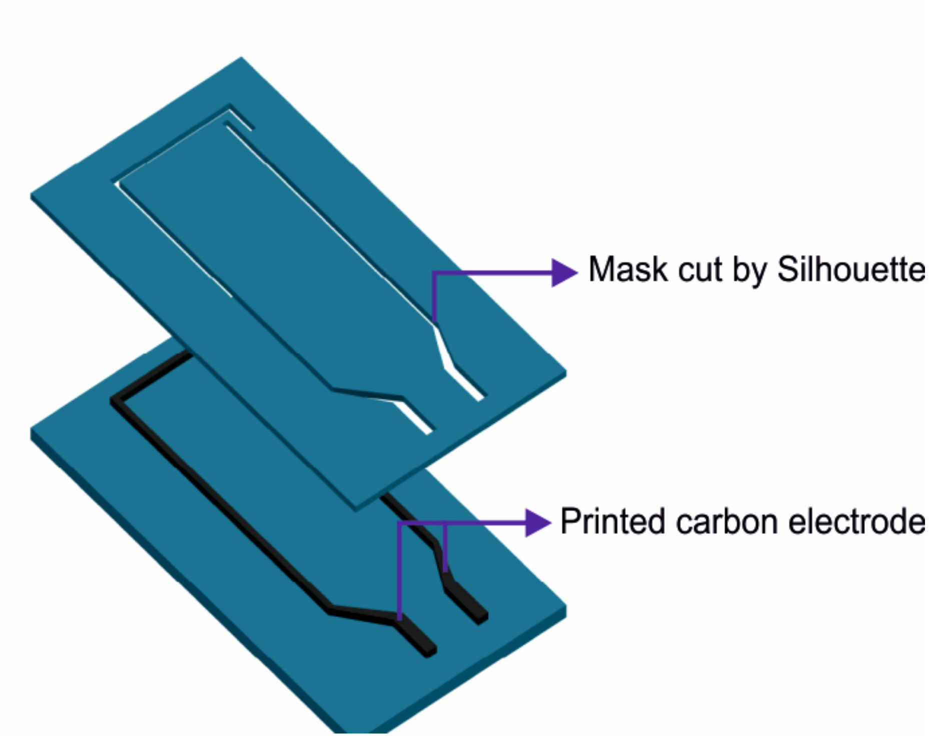

Figure S1. Layering of the screen-printed electrodes included in the sweat-rate-belt-device.

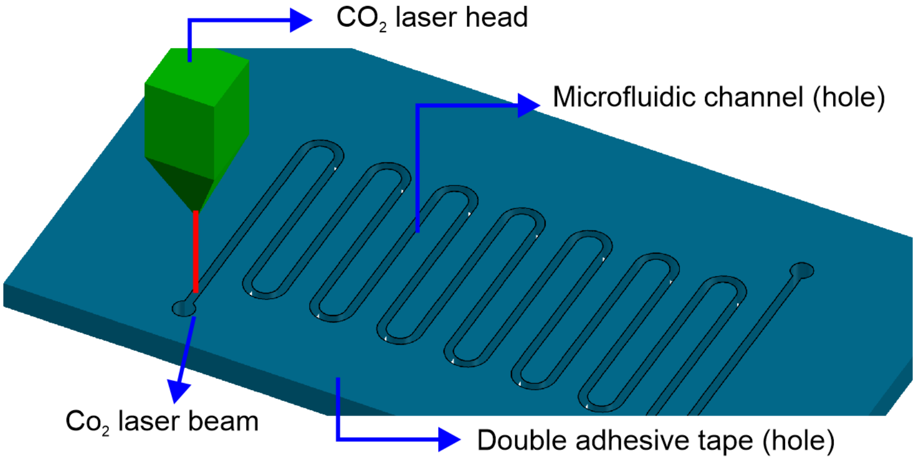

Figure S2. Schematics of the cutting process used to develop the microfluidic channel using the CO_2 laser system.

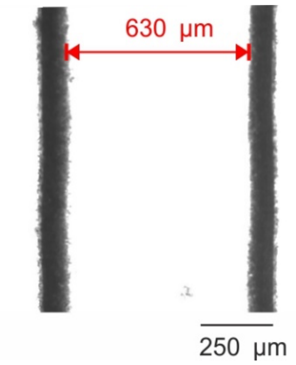

Figure S3. The photo shows the width of microfluidic channel (scale bar is 250 μm).

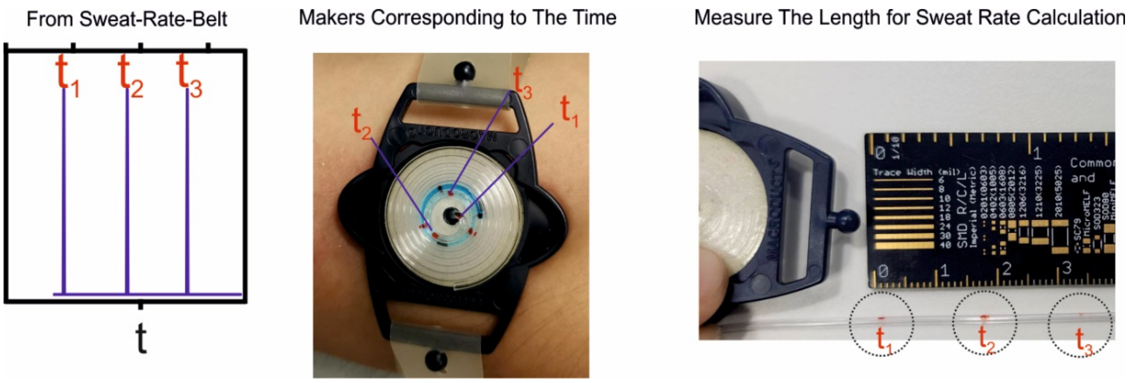

Figure S4. Illustration of the steps included in the method for sweat rate measurement using the Macroduct collector.

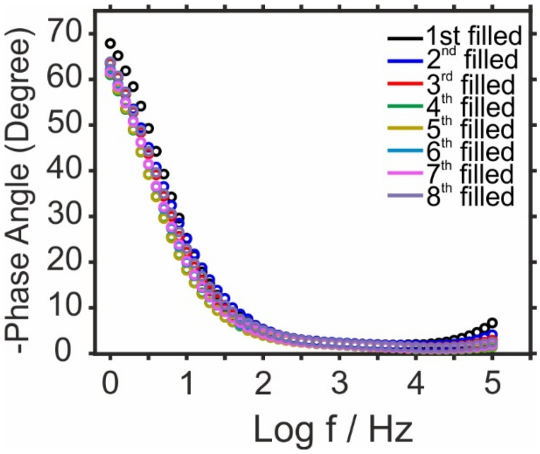

Figure S5. The Electrochemical Impedance Spectroscopy (EIS) performed across a frequency range of 100 kHz to 1 Hz at the different stages of the device filling with artificial sweat.

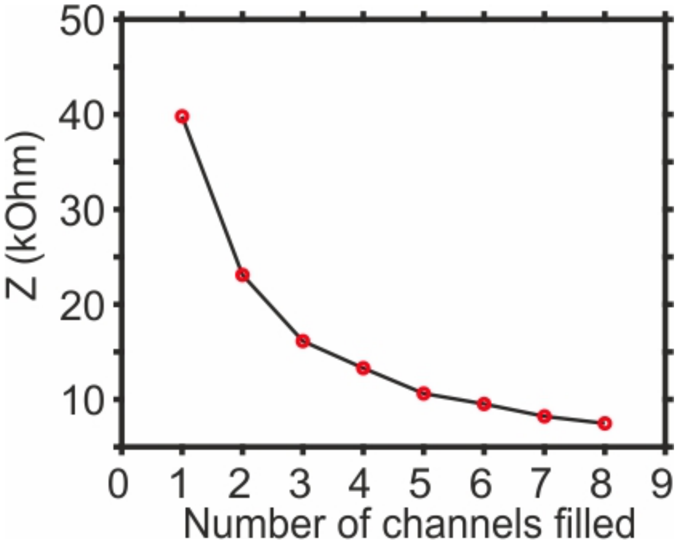

Figure S6. The Impedance value with number of channels filled at 10 kHz.

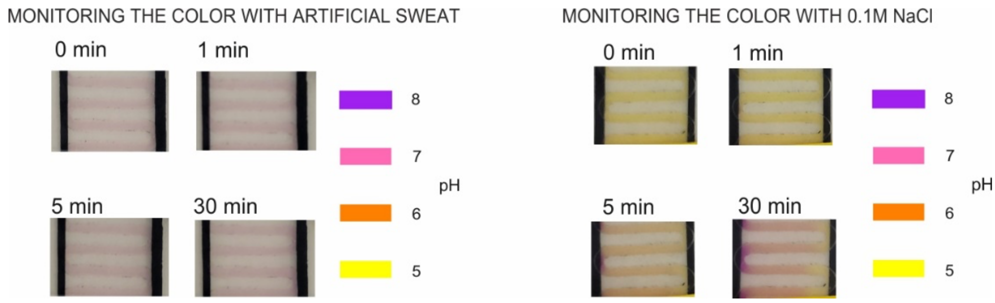

Figure S7. Photographs at 0, 1, 5, and 30 minutes in artificial sweat (left) and 0.1M NaCl (right) after continuously applying the DC current in the system.

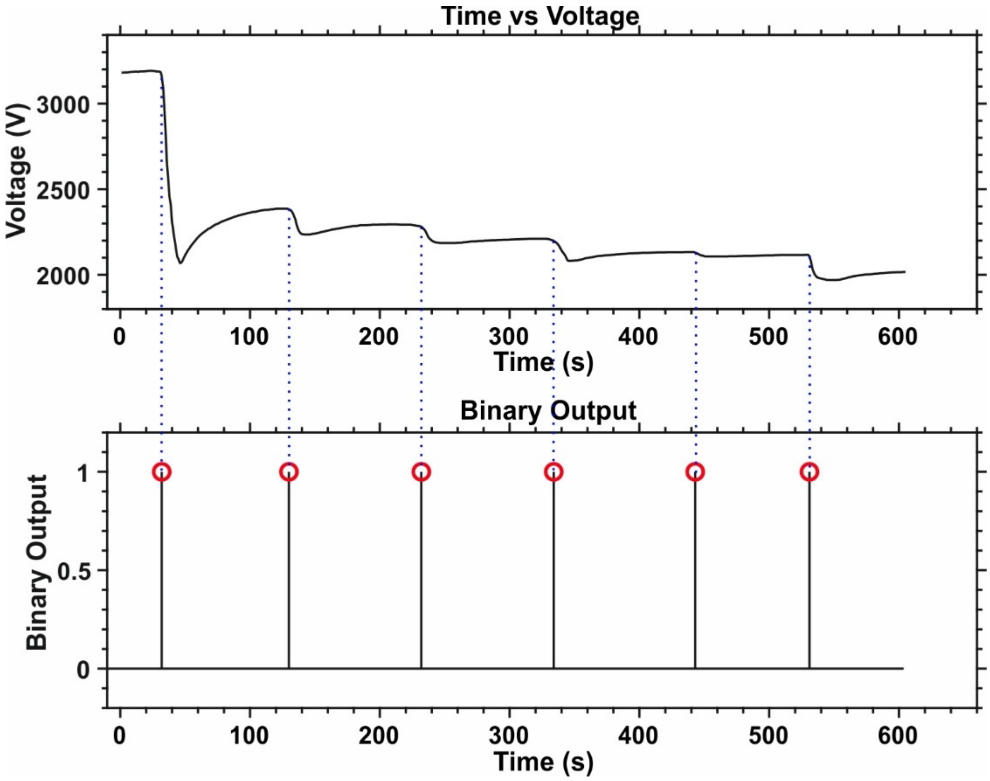

Figure S8. Example of data conversion by transforming a voltage signal into a binary output.

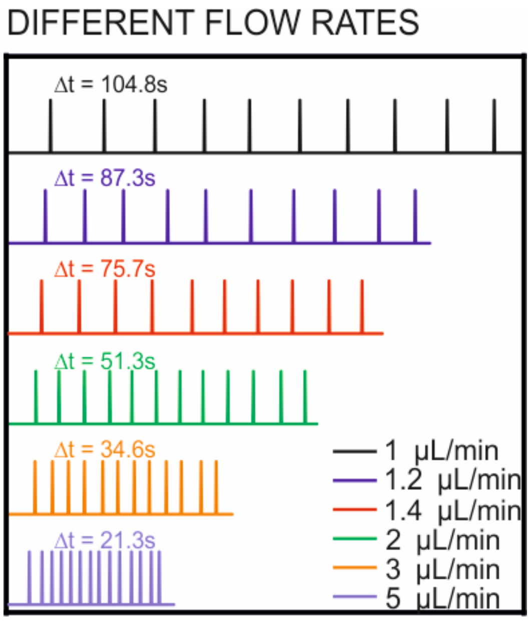

Figure S9. Variation of the binary output at different sample rates. ***Δt*** represents the average time difference between two signal spikes.

2. Tables

Table S1. Comparison of the sweat rate sensors already reported in the literature and that herein developed.

Tech.	Concentration dependent test?	Sweat volume capacity (µL)	Sensor range (µL min^{-1})	Acquisition frequency in the range (Hz)	Validation ?	Microfluidics ?	On-body test?	Readout system?	Ref
Resistance	Yes	7	0.6 – 10	0.06 – 0.1	No	Yes	Yes	Yes	[1]
Resistance	Yes	–	0.1 – 8.0	0.006 – 0.05	No	Yes	Yes	Yes	[2]
Resistance	No	7	1.4 – 3.0	–	No	Yes	Yes	Yes	[3]
Resistance	Yes	15	0.5 – 3.0	0.005 – 0.03	No	Yes	Yes	No	[4]
Colorimetric	No	130	0.6 – 4.0	–	Yes	Yes	Yes	Yes	[5]
Colorimetric	No	130	0.025 – 900	–	No	Yes	Yes	No	[6]
Capacitive	No	130	0.5 – 1.5	–	Yes	Yes	Yes	No	[7]
Capacitive	No	130	0.6 – 23	0.008 – 0.008	No	Yes	Yes	No	[8]
Resistance	Yes	16	1.0 – 5.0	0.01 – 0.043	Yes	Yes	Yes	Yes	This work

Table S2. The error estimation of the Macroduct device using syringe pump. Samples were collected at one-minute intervals during each injection.

**Number of Device**	**test**	**Rate (µL min^{-1})**					
		Injection rate	Macroduct results	Injection rate	Macroduct results	Injection rate	Macroduct results
Device 1	1	5.0	5.1	3.0	2.9	1.0	1.1
	2	5.0	5.0	3.0	3.2	1.0	1.1
	3	5.0	5.1	3.0	3.1	1.0	1.0
	4	5.0	5.3	3.0	3.1	1.0	1.1
	5	5.0	5.3	3.0	2.9	1.0	1.1
	6	5.0	5.1	3.0	2.9	1.0	1.1
Device 2	1	5.0	5.3	3.0	3.2	1.0	1.1
	2	5.0	5.1	3.0	2.9	1.0	1.0
	3	5.0	5.1	3.0	3.2	1.0	1.1
	4	5.0	4.8	3.0	3.4	1.0	1.1
	5	5.0	5.0	3.0	2.9	1.0	1.1
	6	5.0	4.8	3.0	3.2	1.0	1.0
Device 3	1	5.0	5.1	3.0	3.1	1.0	1.1
	2	5.0	5.1	3.0	3.2	1.0	1.0
	3	5.0	5.1	3.0	3.1	1.0	1.1
	4	5.0	5.3	3.0	3.4	1.0	1.1
	5	5.0	5.1	3.0	3.2	1.0	1.0
	6	5.0	5.3	3.0	3.1	1.0	1.1
**Mean**		5.0	5.14	3.0	3.10	1.0	1.08

Table S3. Validation of on-body sweat rate measurements observed with the sweat-rate-belt-device. The results are compared with the Macroduct sweat collector.

Subject	Body parts	Peaks	Sweat rate (μL min^{-1} cm^{-2})		Difference Belt–Macroduct (%)
			Belt	Macroduct	
**#1**	Left arm	1 - 2	2.8	2.5	10.7
	Left arm	2 - 3	3.1	3.4	-9.7
	Left arm	3 - 4	3.4	3.1	8.8
	Left arm	4 - 5	3	2.7	10.0
	Left arm	5 - 6	4.3	4.2	2.3
	Left arm	6 - 7	3.9	4.2	-7.7
	Left arm	7 - 8	4.3	3.9	9.3
	Left arm	8 - 9	3.2	2.9	9.4
	Right arm	1 - 2	2.2	2.4	-9.1
	Right arm	2 - 3	2.3	2.1	8.7
	Right arm	3 - 4	2.0	2.2	-10.0
	Right arm	4 - 5	1.9	1.7	10.5
	Right arm	5 - 6	2.0	2.3	-15.0
	Right thigh	1 - 2	1.4	1.2	14.3
	Right thigh	2 - 3	1.2	1.4	-16.7
	Right thigh	3 - 4	1.4	1.3	7.1
	Right thigh	4 - 5	1.1	1.2	-9.1
**#2**	Left arm	1 - 2	1.2	1.1	8.3
	Left arm	2 - 3	0.9	0.9	0.0
	Right arm	1 - 2	1.8	1.4	22.2
	Right arm	2 - 3	1.8	1.5	16.7
	Right arm	3 - 4	2.3	2.5	-8.7
	Right arm	4 - 5	2.1	2.1	0.0
	Right arm	5 - 6	2.2	2.0	9.1
	Right arm	6 - 7	2.3	2.2	4.3

3. MATLAB script

Script S1. MATLAB script of the algorithm for convert time vs. voltage to time vs. binary.

```
function binary_output = time_vs_voltage_to_binary(time, voltage, interval,
delta_threshold)
    % time: raw data of time
    % voltage: raw data of voltage
    % interval: time interval to check (e.g., 1 second)
    % delta_threshold: threshold for voltage drop

    % Initialize binary output array with zeros
    binary_output = zeros(size(time));

    % Get total length of the time array
    N = length(time);

    % Starting index for checking
    start_index = 1;

    % Iterate through time array with intervals
    while start_index < N
        % Set the end index for the current interval
        end_index = find(time >= time(start_index) + interval, 1);
        if isempty(end_index)
            end_index = N; % If there is no exact interval, check till the end
        end

        % Look for the first significant drop in voltage within the current
interval
        for i = start_index+1:end_index
            delta_voltage = voltage(i-1) - voltage(i);
            if delta_voltage >= delta_threshold
                % Mark this time point as 1 in binary output
                binary_output(i) = 1;

                % Move the start index forward by the interval
                start_index = find(time >= time(i) + interval, 1);
                if isempty(start_index)
                    start_index = N; % If no interval, finish the process
                end
                break; % Move to the next interval once we find a valid drop
            end
        end

        % If no voltage drop found in the interval, move to the next interval
        if start_index < end_index
            start_index = end_index;
        end
    end
end
```

4. REFERENCES

(1) Lin, H.; Yu, W.; Suarez, J. E. D. D.; Athavan, H.; Wang, Y.; Yeung, C.; Lin, S.; Sankararaman, S.; Milla, C.; Emaminejad, S. Autonomous wearable sweat rate monitoring based on digitized microbubble detection. *Lab on a Chip* **2022**, *22* (22), 4267-4275.
(2) Wei, L.; Lv, Z.; He, Y.; Cheng, L.; Qiu, Y.; Huang, X.; Ding, C.; Wu, H.; Liu, A. In-situ admittance sensing of sweat rate and chloride level in sweat using wearable skin-interfaced microfluidic patch. *Sensors and Actuators B: Chemical* **2023**, *379*, 133213.
(3) Yang, Q.; Rosati, G.; Abarintos, V.; Aroca, M. A.; Osma, J. F.; Merkoçi, A. Wearable and fully printed microfluidic nanosensor for sweat rate, conductivity, and copper detection with healthcare applications. *Biosensors and Bioelectronics* **2022**, *202*, 114005.
(4) Yuan, Z.; Hou, L.; Bariya, M.; Nyein, H. Y. Y.; Tai, L.-C.; Ji, W.; Li, L.; Javey, A. A multi-modal sweat sensing patch for cross-verification of sweat rate, total ionic charge, and Na+ concentration. *Lab on a Chip* **2019**, *19* (19), 3179-3189.
(5) Baker, L. B.; Model, J. B.; Barnes, K. A.; Anderson, M. L.; Lee, S. P.; Lee, K. A.; Brown, S. D.; Reimel, A. J.; Roberts, T. J.; Nuccio, R. P. Skin-interfaced microfluidic system with personalized sweating rate and sweat chloride analytics for sports science applications. *Science advances* **2020**, *6* (50), eabe3929.
(6) Wang, H.; Xu, K.; Xu, H.; Huang, A.; Fang, Z.; Zhang, Y.; Wang, Z. e.; Lu, K.; Wan, F.; Bai, Z. A One-Dollar, Disposable, Paper-Based Microfluidic Chip for Real-Time Monitoring of Sweat Rate. *Micromachines* **2022**, *13* (3), 414.
(7) Choi, D.-H.; Gonzales, M.; Kitchen, G. B.; Phan, D.-T.; Searson, P. C. A capacitive sweat rate sensor for continuous and real-time monitoring of sweat loss. *ACS sensors* **2020**, *5* (12), 3821-3826.
(8) Sim, J. K.; Yoon, S.; Cho, Y.-H. Wearable sweat rate sensors for human thermal comfort monitoring. *Scientific reports* **2018**, *8* (1), 1181.
